# NR1I2 genetic polymorphisms and the risk of anti‐tuberculosis drug‐induced hepatotoxicity: A systematic review and meta‐analysis

**DOI:** 10.1002/prp2.696

**Published:** 2020-12-10

**Authors:** Miaomiao Yang, Yunliang Qiu, Yanyu Jin, Wenpei Liu, Qingliang Wang, Honggang Yi, Shaowen Tang

**Affiliations:** ^1^ Department of Epidemiology and Biostatistics School of Public Health Nanjing Medical University Nanjing China; ^2^ Department of Criminal Science and Technology Nanjing Forest Police College Nanjing China; ^3^ School of Pediatrics Nanjing Medical University Nanjing China; ^4^ Department of Medical Affairs Qilu Hospital of Shandong University Jinan China

**Keywords:** anti‐tuberculosis drug‐induced hepatotoxicity, genetic polymorphisms, meta‐analysis, NR1I2, pregnane X receptor

## Abstract

Anti‐tuberculosis drug‐induced hepatotoxicity (ATDH) is a serious adverse drug reaction. Conflicting results have been obtained regarding the associations of nuclear receptor subfamily 1 group I member 2 (NR1I2) gene polymorphisms on susceptibility to ATDH. Therefore, we aimed to evaluate the associations using a systematic review/meta‐analysis approach. PubMed, Medline, Cochrane Library, Web of Science and SinoMed databases were searched for all eligible studies from inception to June 10, 2020. Pooled adjusted odds ratios (ORs) with 95% confidence intervals (CIs) were employed to evaluate the strength of the association between the NR1I2 polymorphisms and the risk of ATDH. Subgroup analysis was performed by region of origin, and meta‐regression were performed to detect potential sources of heterogeneity. A total of five case‐control studies involving 572 cases and 1867 controls were identified. Fourteen SNPs in the NR1I2 gene have been reported, and the most heavily studied SNPs were rs3814055 and rs7643645. The pooled estimates did not exhibit any significant associations between SNPs rs3814055 and rs7643645 and the risk of ATDH (rs3814055: dominant model, OR = 1.00, 95% CI: 0.82‐1.22, *P* = 1.00; recessive model, OR = 1.17, 95% CI: 0.76‐1.78, *P* = .48; rs7643645: dominant model, OR = 1.04, 95% CI: 0.64‐1.68, *P* = .89; recessive model, OR = 0.98, 95% CI: 0.65‐1.49, *P* = .93). Subgroup analysis obtained similar negative results in Chinese patients, and the diagnostic criteria of ATDH may be the source of heterogeneity. Based on the meta‐analysis described in this report, we did not observe any association between NR1I2 gene polymorphisms and ATDH susceptibility. However, this conclusion should be interpreted with caution due to the low number of studies and the relatively small sample size.

## Graphical abstract

Based on the systematic review and meta‐analysis with five case‐control studies, no significant association was observed between genetic polymorphisms of NR1I2 and the risk of anti‐tuberculosis drug‐induced hepatotoxicity.

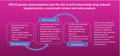

**Abbreviations**
ADR: adverse drug reaction
ALAS1: aminolevulinic synthase‐1
ATDH: anti‐tuberculosis drug‐induced hepatotoxicity
CI: confidence interval
CYP450: cytochrome P450
DILI: drug‐induced liver injury
EMB: ethambutol
FECH: ferrochelatase
INH: isoniazid
NR1I2: nuclear receptor subfamily 1 group I member 2
OR: odds ratio
PPⅨ: protoporphyrin Ⅸ
PXR: pregnane X receptor
PZA: pyrazinamide
RIF: rifampicin
RUCAM: Roussel Uclaf Causality Assessment Method
SinoMed: Chinese Biomedical Literature Service System
SNP: single‐nucleotide polymorphism
TB: tuberculosis
WHO: World Health Organization

## 1 INTRODUCTION

Tuberculosis (TB) is a chronic infectious disease caused by *Mycobacterium tuberculosis* (Mtb); this disease remains a major cause of death and suffering worldwide, and its control is a global public health issue.^1^ The Global Tuberculosis Report 2019 released by the World Health Organization (WHO) indicated that there were 10 million new cases and 1.5 million deaths in 2018, and China had the second largest number of new TB cases worldwide with 886,000 estimated new cases and an incidence of 61/100,000.^2^ The treatment of TB with short‐course chemotherapy, recommended by the WHO TB program, has remained largely unchanged for the past 40 years; specifically, with this treatment, a combination of isoniazid (INH), rifampicin (RIF), pyrazinamide (PZA), ethambutol (EMB), and streptomycin is administered for a period of 6‐8 months.^3^ Although chemotherapy has dramatically increased the effectiveness of TB control, achieving a treatment success rate of 85%,^2^ a variety of adverse drug reactions (ADRs) may occur during long‐term multidrug combination therapy, among which anti‐tuberculosis drug‐induced hepatotoxicity (ATDH) is the most common adverse event that necessitates therapy interruption.^4^ The manifestations of ATDH may vary from asymptomatic elevations in the liver enzymes to fulminant liver failure.^5^ ATDH is also a common cause of drug‐induced liver injury (DILI) in China.^6^

Although the pathogenic mechanism underlying ATDH has not been fully elucidated, a number of hypotheses on the pathogenesis of ATDH have been proposed, such as drug metabolism and transporter enzymes, immune response, oxidative stress, and mitochondrial dysfunction.^7^, ^8^ Among the first‐line anti‐TB drugs, INH and RIF have been demonstrated to cause hepatotoxicity.^9^ Recently, based on a pregnane X receptor (PXR)‐humanized mouse model, Li and colleagues made an outstanding and substantial contribution towards elucidating the mechanism of ATDH by determining that cotreatment with RIF and INH causes accumulation of the endogenous hepatotoxin protoporphyrin Ⅸ (PPⅨ) in the liver through PXR‐mediated alteration of the heme biosynthesis pathway.^10^ PPIX is ubiquitous in all living cells in small amounts as a precursor of heme, and accumulation of PPIX in live human cells can cause hepatobiliary damage, liver injury, and even liver failure.^11^, ^12^ The RIF‐INH co‐therapy caused the accumulation of PPIX through PXR‐mediated transcriptional activation of both the cytochrome P450 (CYP450) and aminolevulinic synthase‐1 (ALAS1) genes.^13^ ALAS1 is the rate‐limiting enzyme in the production of heme in the liver and is upregulated by RIF‐INH therapy, both by direct transcriptional activation and through derepression of negative feedback due to the incorporation of heme into the CYP450 apoprotein.^10^ INH was determined to downregulate ferrochelatase (FECH), the enzyme that converts PPⅨ to heme, and to cause PPⅨ accumulation and liver injury.^12^ FECH inhibition and ALAS1 induction may exert a synergistic effect on PPIX accumulation.^14^ All these findings help to elucidate the mechanism underlying RIF and INH co‐therapy‐induced liver injury, which may be applied to study the risk factors and genetic susceptibility to ATDH, as well as the prevention and control of liver injury.

PXR, encoded by the nuclear receptor subfamily 1 group I member 2 (NR1I2) gene, is a ligand‐dependent transcription factor that is involved in the gene network regulating the metabolism of exogenous and endogenous substances.^15^ Based on the PubMed Gene website (www.ncbi.nlm.nih.gov/gene), the human NR1I2 gene on chromosome 3q13.33 comprises ten exons, spans approximately 38 kilobases, encodes 434 amino acids, and contains 11 054 single‐nucleotide polymorphisms (SNPs) (GRCh38.p13, Chr3: 119780484‐119818485). Genetic polymorphism could affect gene transcription and the activity of proteins encoded, which in turn may lead to changes in the pharmacokinetic and pharmacodynamic behavior of a drug, observed as differences in drug trans port, drug metabolism, pharmacodynamic drug effects, and adverse events.^16^ Similarly, NR1I2 genetic polymorphisms can affect the pharmacokinetics and therapeutic response to many drugs, such as irinotecan, tacrolimus and atazanavir.^17^ Rana and colleagues found that some of the non‐synonymous variants of PXR may have adverse physiological consequences owing to its influence on the expression levels and functional output of drug‐metabolizing enzymes and transporters,^18^ and the T‐allele was associated with significantly greater transcriptional activity than the C‐allele of SNP rs3814055.^19^ To date, a number of studies have been conducted to investigate the potential association between NR1I2 genetic polymorphisms and the risk of ATDH, with inconsistent results being obtained.^20^, ^21^, ^22^, ^23^, ^24^ For example, earlier studies from Indonesia showed that patients with the TT genotype at rs3814055 had a significantly increased risk of ATDH,^21^ but another study from China reached the opposite conclusion, namely, the T allele of rs3814055 was associated with a decreased risk for ATDH.^20^ However, a systematic review and meta‐analysis can be used to pool results from different studies, thereby enhancing the precision of estimates of treatment effects.^25^ To the best of our knowledge, no systematic review or meta‐analysis has been undertaken to clarify the effect of these polymorphisms on the risk of ATDH. So, we aimed to evaluate the association between NR1I2 genetic polymorphisms and the risk of ATDH using a systematic review/meta‐analysis approach, and provide more accurate conclusions regarding genetic susceptibility research on ATDH and identify areas that warrant further investigation.

## 2 MATERIALS AND METHODS

### 2.1 Search strategies

This systematic review and meta‐analysis followed the PRISMA statement and guidelines.^26^ The literature search was performed in the PubMed, Medline, Cochrane Library, Web of Science and SinoMed (Chinese Biomedical Literature Service System) databases from inception to June 10, 2020, for all relevant papers, and the search terms included “PXR” or “NR1I2” or “pregnane X receptor” or “nuclear receptor subfamily 1 group I member 2”, “drug‐induced liver injury” or “drug‐induced hepatotoxicity” or “drug‐induced hepatitis” or “drug‐induced liver damage” or “drug‐induced hepatic injury” or “toxic hepatitis,” and “antituberculosis” or “anti‐tuberculosis” or “antitubercular” or “tuberculosis treatment.” The work was updated before the statistical analysis was performed to prevent the latest published relative report from being lost. The full search strategies for each database are listed in the Supplementary Material (Table S1).

All the records identified from the databases mentioned above have been imported into EndNote X8 (Thomson Reuters, New York, NY), and duplicate records have been deleted. Reviewers were divided into two groups that worked in parallel. The reviewers independently screened each record by title, keywords, and abstract against the eligibility criteria. Full texts were referred to when information in the records was inadequate for determination. Any disagreement between the two groups of reviewers was resolved by an additional reviewer. Manual searching was performed by reviewing the references of the included studies.

### 2.2 Inclusion and exclusion criteria

The eligible studies included in the present study met the following criteria: (a) case‐control studies to assess the association between NR1I2 polymorphisms and risk of ATDH were analyzed; (b) cases were TB patients with ATDH, while controls were TB patients without ATDH; (c) TB patients receiving first‐line anti‐TB drug treatment were investigated; (d) studies reported odds ratios (ORs) with 95% confidence intervals (CIs) for the risk of ATDH or sufficient data to estimate ORs and their 95% CIs; and (e) the language was restricted to English or Chinese.

The studies were excluded if (a) they were conference abstracts, protocols, or summaries; (b) the sample size for each group was less than 10; and (c) studies with duplicate data were reported in multiple studies by the same research group.

### 2.3 Data extraction

An extraction form was designed to extract data, and the following information was extracted from each study if available: (a) study characteristics: first author, publication year, study design, inclusion and exclusion criteria, and sample size; (b) population characteristics: sex, mean/median age of total subjects, treatment regimen used, diagnostic criteria of ATDH, method of causality assessment, and covariates adjusted; and (c) adjusted ORs with 95% CIs under different genetic models reported by the original study or allele frequencies in ATDH cases and controls. The data extraction procedure was also implemented independently by the two parallel groups of reviewers. Any disagreement was resolved by an additional reviewer. All data were directly taken from the included studies, and no further information was obtained by consulting the authors.

### 2.4 Quality assessment

Study quality was assessed by the following revised criteria according to Little's recommendations^27^ to determine potential bias and its effect on summary results. These criteria included seven items: (a) scientific design, (b) definite inclusion of study population, (c) explicit information on study population, (d) explicit diagnostic criteria on ATDH, (e) genetic detection method, (f) correct statistical analysis and (g) logical discussion of study bias. Each item can be judged as “yes” (low risk of bias) or “no” (high risk of bias). One point was awarded if an item was judged as low risk of bias. An overall quality scoring was generated, with a maximum score of 7 points, and a score > 4 was defined as a study of high quality.^28^

### 2.5 Statistical analysis

Pooled adjusted ORs with 95% CIs were employed to evaluate the strength of association between the NR1I2 polymorphisms and the risk of ATDH, and two genetic models were employed to calculate their associations, including dominant (MW + MM vs. WW) and recessive models (MM vs. WW + WW) (W refers to a wild‐type allele, and M refers to a mutated allele). Additionally, the pooled ORs were estimated for each NR1I2 polymorphism based on the allele comparison (M vs. W). The significance of pooled ORs was defined as *P* < .05 from the Z test, and heterogeneity across studies was determined by the Cochran Q statistic and the I^2^ test.^29^ When I^2^ ≤ 50% or *P* ≥ .1, the heterogeneity was overlooked; next, the fixed‐effect model was applied; otherwise, the random‐effect model was selected. Subgroup analyses was performed by region of origin. Moreover meta‐regression was employed to reveal whether diagnostic criteria of ATDH, causality assessment, or adjustment of covariates could lead to heterogeneity. This meta‐analysis was performed using Review Manager version 5.3.5 (the Nordic Cochrane Center, Copenhagen, Denmark). Meta‐regression analysis was performed with the metafor package based on R software for Windows version 3.5.3 (https://www.r‐project.org/).

## 3 RESULTS

### 3.1 Study identification and characteristics

**Figure 1:**
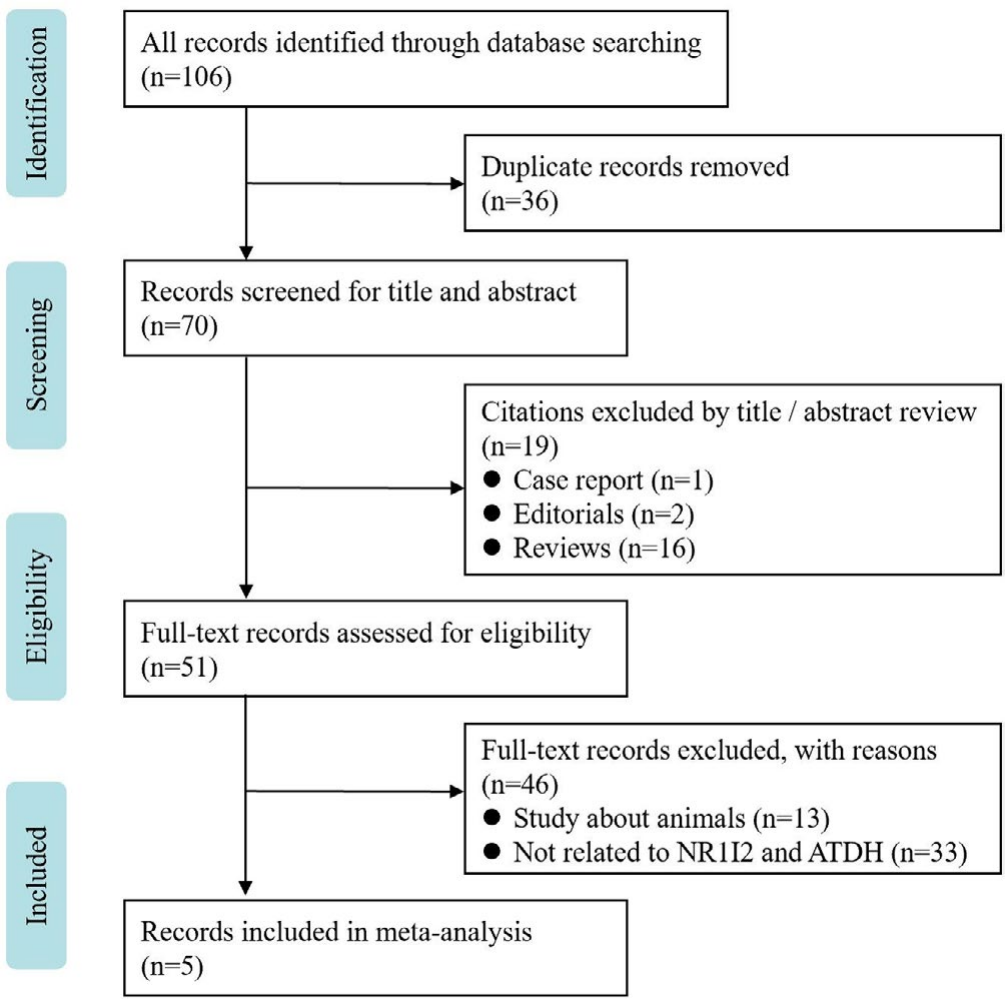
PRISMA flow chart for literature search

A total of 106 relevant articles were identified after an initial screening, and 5 case‐control studies, which consisted of 572 ATDH cases and 1867 controls, were included.^20^, ^21^, ^22^, ^23^, ^24^ The flow chart of the included and excluded studies is shown in Figure 1. There were 14 SNPs in the NR1I2 gene that have been reported previously (ie, rs3814055, rs7643645, rs13059232, rs2461823, rs3814057, rs6785049, rs12488820, rs1523127, rs2276707, rs2461825, rs2472677, rs2472682, rs3732357, and rs3732360), and the most heavily studied SNPs were rs3814055 and rs7643645. Table 1 lists the included studies and their primary characteristics. Included studies covered Chinese^20^, ^22^, ^23^, ^24^ and Indonesian,^21^ and different criteria for ATDH diagnosis were employed in the enrolled studies; these criteria included those of the DILI Network,^21^ the International Consensus Meeting,^23^ the American Thoracic Society,^22^ the National Institutes of Health and the Common Toxicity Criteria for Adverse Events,^20^ and the Chinese Society of Hepatology.^24^ Only one study^24^ performed a causality assessment using the updated Roussel Uclaf Causality Assessment Method (RUCAM).^30^ The average quality score was 6.2, which demonstrated that the methodological quality was better.

**Table 1:** Characteristics of the studies included in the meta‐analysis

Study ID	Country	Study design	Male (%)	Age (years)	Sample size (Cases/Controls)	Causality assessment	Diagnostic criteria	SNPs	Genotyping method	Adjusted covariates	Quality score
Zazuli 2015	Indonesia	Nested case‐control	67.9	NR	35/71	NR	>1 ULN	rs3814055	PCR‐ARMS	Sex, age	6
Wang 2015	China Taiwan	Nested case‐control	65.6	57.6	70/285	NR	>3 ULN	rs3814055, rs12488820, rs2461823, rs7643645, rs6785049, rs3814057	Sequenom MassARRAY	NR	6
Wang 2019	China	Case‐control	49.4	38.5	203/299	NR	>2 ULN	rs7643645, rs6785049, rs3732357, rs3814055, rs2472682, rs3814057, rs2472677	iMLDR	Ethnicity, age, gender, height, weight, smoking, drinking, HbsAg status	6
Zhang 2019	China	Case‐control	59.5	41.2	118/628	NR	>3 ULN	rs3814055, rs13059232, rs7643645, rs3732360	SNPscan	Sex, age	6
Yang 2020	China	1:4 matched case‐control^a^	73.3	50.4	146/584	RUCAM	>3 ULN	rs2276707, rs3814055, rs1523127, rs13059232, rs2461823, rs2461825, rs7643645	TaqMan	Liver diseases history, hepatoprotectant use, smoking history, drinking history	7

Abbreviations: iMLDR, improved multiplex ligation detection reaction technique; NR, Not Report; PCR‐ARMS, polymerase chain reaction–amplification refractory mutation system; RUCAM, Roussel Uclaf Causality Assessment Method; SNPs, single‐nucleotide polymorphisms; ULN, Upper Limit of Normal.

^a^ Matched with age (within 5 years old), sex, and treatment history.

### 3.2 Quantitative analysis

**Figure 2:**
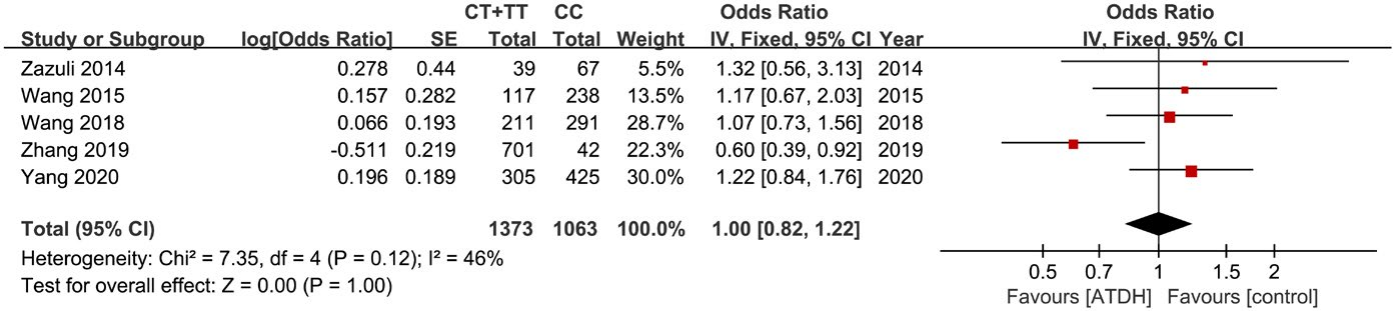
Forest plot of the relation between SNP rs3814055 (dominant model) and the risk of ATDH with the fixed effects model

**Figure 3:**
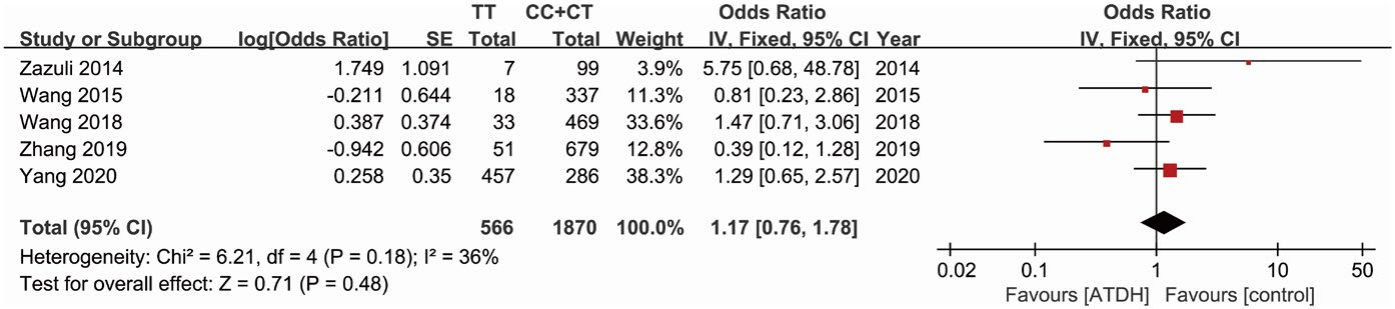
Forest plot of the relation between SNP rs3814055 (recessive model) and the risk of ATDH with the fixed effects model

Five studies all explored the relationship between SNP rs3814055 and ATDH susceptibility.^20^, ^21^, ^22^, ^23^, ^24^ Using the fixed‐effects model, the pooled estimates of the five included studies did not show a significant association between SNP rs3814055 and the risk of ATDH (dominant model, OR = 1.00, 95% CI: 0.82‐1.22, *P* = 1.00, Figure 2; recessive model, OR = 1.17, 95% CI: 0.76‐1.78, *P* = .48, Figure 3) (Table 2).

**Table 2:** Meta‐analysis results of the association between gene polymorphism of NR1I2 and the susceptibility of ATDH

Patients source	SNPs	Study numbers	Dominant model					Recessive model				
			Heterogeneity test			Overall effect		Heterogeneity test			Overall effect	
			χ^2^	P‐value	I^2^ (%)	OR(95%CI)	P‐value	χ^2^	P‐value	I^2^ (%)	OR(95%CI)	P‐value
All patients	rs3814055	5	7.35	0.12	46	1.00(0.82‐1.22)	1.00	6.21	0.18	36	1.17(0.76‐1.78)	0.48
	rs7643645	4	13.13	0.01	77	1.04(0.64‐1.68)	0.89	8.55	0.04	65	0.98(0.65‐1.49)	0.93
	rs13059232	2	2.20	0.14	55	1.09(0.82‐1.43)	0.56	0.06	0.80	0	1.18(0.82‐1.70)	0.37
	rs2461823	2	0.02	0.88	0	1.25(0.91‐1.73)	0.17	4.93	0.03	80	0.73(0.30‐1.78)	0.49
	rs3814057	2	0.13	0.72	0	1.36(0.93‐1.98)	0.11	0.87	0.38	0	0.85(0.59‐1.22)	0.38
	rs6785049	2	0.00	0.98	0	1.21(0.87‐1.68)	0.25	3.13	0.08	68	0.99(0.42‐2.32)	0.97
Chinese patients	rs3814055	4	6.92	0.07	57	0.98(0.71‐1.35)	0.89	3.98	0.26	25	1.09(0.71‐1.68)	0.69

Abbreviations: 95%CI, 95% confidence interval; ATDH, anti‐tuberculosis drug‐induced hepatotoxicity; NR1I2, nuclear receptor subfamily 1 group I member 2; OR, Odds ratio; SNPs, single‐nucleotide polymorphisms.

**Figure 4:**
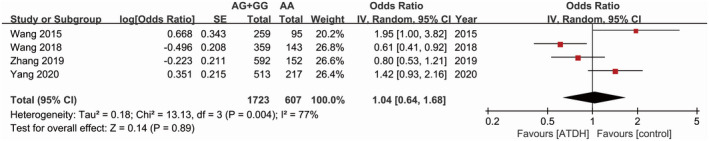
Forest plot of the relation between SNP rs7643645 (dominant model) and the risk of ATDH with the random effects model

**Figure 5:**
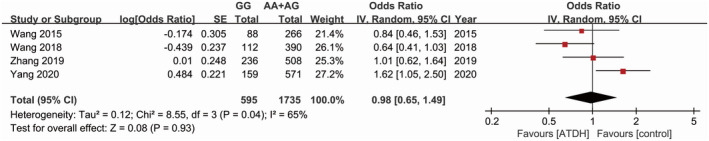
Forest plot of the relation between SNP rs7643645 (recessive model) and the risk of ATDH with the random effects model

Four studies with 537 cases and 1796 controls investigated the effect of SNP rs7643645 on the risk of developing ATDH,^20^, ^22^, ^23^, ^24^ and all patients were Chinese. Using the random‐effect model, the pooled estimates of four included studies also did not indicate a significant association between SNP rs7643645 and the risk of ATDH (dominant model, OR = 1.04, 95% CI: 0.64‐1.68, *P* = .89, Figure 4; recessive model, OR = 0.98, 95% CI: 0.65‐1.49, *P* = .93, Figure 5) (Table 2).

Four SNPs (rs13059232, rs2461823, rs3814057, rs6785049) were reported by two different studies, and the adjusted ORs were pooled by different models. No further significant association was observed between these SNP polymorphisms and ATDH susceptibility (Table 2, Figure S1‐S8). The remaining 8 SNPs (ie, rs12488820, rs1523127, rs2276707, rs2461825, rs2472677, rs2472682, rs3732357, and rs3732360) reported only in a single original study were all from Chinese patients,^20^, ^22^, ^23^, ^24^ and no genotypes were found to be significantly related to ATDH, except one SNP, rs2276707, under the recessive model (OR = 0.600, 95% CI: 0.364‐0.988, *P* = .045).^24^

Additionally, there was no evidence for a significant association between six SNPs (ie, rs3814055, rs7643645, rs13059232, rs2461823, rs3814057, and rs6785049) and the risk of ATDH in the allele comparison model (Table 3).

**Table 3:** Meta‐analysis results of the association between six SNPs in NR1I2 and the risk of ATDH in allele comparison models

SNPs	Study numbers	Heterogeneity test			Overall effect	
		χ^2^	*P*‐value	I^2^(%)	OR(95% CI)	*P*‐value
rs3814055 (T vs. C)	5	10.73	0.03	63	1.03(0.77‐1.37)	0.84
rs7643645 (G vs. A)	4	16.54	<0.001	82	1.03(0.74‐1.45)	0.85
rs13059232 (C vs. T)	2	1.33	0.25	25	1.09(0.90‐1.32)	0.40
rs2461823 (C vs. T)	2	0.67	0.41	0	1.21(0.98‐1.50)	0.08
rs3814057 (C vs. A)	2	0.20	0.65	0	1.02(0.83‐1.26)	0.83
rs6785049 (G vs. A)	2	0.00	0.95	0	0.98(0.79‐1.21)	0.83

Abbreviations: 95% CI, 95% confidence interval; ATDH, anti‐tuberculosis drug‐induced hepatotoxicity; NR1I2, nuclear receptor subfamily 1 group I member 2; OR, Odds ratio; SNPs, single‐nucleotide polymorphisms.

### 3.3 Subgroup analysis

The subgroup analysis was conducted to characterize the role played by SNP rs3814055 only in Chinese patients (four studies with 537 cases and 1796 controls). However, no significant evidence of an association was observed between SNP rs3814055 and the risk of ATDH in Chinese patients (dominant model, OR = 0.98, 95% CI: 0.71‐1.35, I^2^ = 57%, *P* = .89; recessive model, OR = 1.09, 95% CI: 0.71‐1.68, *P* = .69) (Table 2).

### 3.4 Meta‐regression analysis

Extended meta‐regression was performed to explore the source of heterogeneity in four studies of the effect of SNP rs7643645, and the results are shown in Table 4. Only the diagnostic criteria of ATDH may explain the source of heterogeneity under the recessive model (R^2^ = 100.00%, *P* = .039), and marginal significance was observed in the causality assessment under the dominant model (R^2^ = 81.56%, *P* = .068).

**Table 4:** Results of univariate meta‐regression analyses of SNP rs7633645 under different models

Model	Covariates	I^2^	R^2^ (%)	*P*
Dominant model	None	0.120	‐	‐
(AG + GG Vs. AA)	Diagnostic criteria of ATDH	0.098	18.29	0.182
	Causality assessment	0.022	81.56	0.068
	Adjustment of covariates	0.104	13.54	0.293
Recessive model	None	0.066	‐	‐
(GG Vs. AA + AG)	Diagnostic criteria of ATDH	0	100.00	0.039
	Causality assessment	0.017	74.44	0.075
	Adjustment of covariates	0.135	0	0.829

Abbreviations: ATDH, anti‐tuberculosis drug‐induced hepatotoxicity; R^2^, amount of heterogeneity accounted for; SNP, single‐nucleotide polymorphism.

## 4 DISCUSSION

The present study investigated the genetic association between the 14 polymorphisms of NR1I2 genes and susceptibility to ATDH. Our study was the first to gather all the case‐control studies performed on those associations. Based on the five studies included in this meta‐analysis, no significant association was observed between SNP rs3814055 and the risk of ATDH after ORs were pooled under dominant and recessive models. Subgroup analysis also reproduced similar negative results in Chinese patients under both models. However, among the five original studies, one showed that patients with the T allele had a decreased risk of ATDH (OR = 0.60, 95% CI: 0.39‐0.92, *P* = .02) under the dominant model,^20^ while another study reported that patients with the TT genotype had a greater risk of ATDH under the codominant model (OR = 8.89, 95% CI: 1.36‐57.93, *P* < .05)^21^; the remaining studies showed no significant association between the rs3814055 genotype and risk of ATDH under three classic genetic models.^22^, ^23^, ^24^ However, previous studies indicated that the change from the C allele to the T allele at SNP rs3814055 may be functional. A cell study showed that the TT genotype of SNP rs3814055 was associated with higher induction of CYP3A4 activity by rifampicin,^31^ and a change from a C allele to a T allele was associated with significantly greater transcriptional activity,^19^ indicating that the SNP rs3814055 C/T polymorphism may have an effect on the transcriptional upregulation of PXR. In another study of flucloxacillin‐induced hepatotoxicity, the CC genotype was associated with an increased risk of hepatocyte injury in the presence of the decreased expression of CYP3A4, which may result in a higher accumulation of unmetabolized toxic drugs and may lead to hepatocellular injury.^32^ Therefore, the SNP rs3814055 polymorphism may be primarily related to CYP3A4 activity. However, based on the present meta‐analysis, none of the obtained ORs showed any relation between SNP rs3814055 and the risk of ATDH.

Based on the four original Chinese studies, we further observed a lack of association between the rs7643645 polymorphism and ATDH susceptibility. However, among the four original studies, Wang reported that the GG genotype at SNP rs7643645 was significantly associated with decreased ATDH risk (dominant model: OR = 0.609, 95% CI: 0.405‐0.917, *P* = .017).^23^ Another study demonstrated that the risk of ATDH decreased in female genotype AA at rs7643645 (OR = 0.14, 95% CI: 0.02‐1.02, *P* = .052),^22^ which is generally consistent with the results of another Chinese patient study (GG vs. AA, OR = 1.622, 95% CI: 1.052‐2.502, *P* = .029).^24^ The difference between those studies may be attributed to multiple factors, such as different diagnostic criteria of ATDH, the use of causality assessment, and the adjusted covariates (Table 1). The meta‐regression analysis showed that the diagnostic criteria of ATDH might be the most likely potential sources of heterogeneity between studies in dominant model analysis (Table 4); one study employed 2 upper limits of normal (ULNs) of elevated liver enzymes,^23^ while others employed 3 ULNs.^20^, ^22^, ^24^ Additionally, marginal significance in meta‐regression was found in causality assessment under the dominant model, and only one study employed the Roussel Uclaf Causality Assessment Method (RUCAM), which is a well‐established tool commonly utilized to quantitatively assess causality in cases of suspected DILI.^33^ However, although significant heterogeneity was observed in the combined analysis, the pooled results still indicated that the SNP rs7643645 polymorphism may not be associated with the risk of ATDH.

Combined with the results obtained with the remaining 4 SNPs, the present meta‐analysis failed to detect any significant association between SNP polymorphisms in the NR1I2 gene and ATDH susceptibility. Indeed, ATDH is a complex disease, and multiple metabolic enzymes and pathways are involved in its pathophysiology.^34^ In the PXR‐mediated alteration of the heme biosynthesis pathway, RIF‐INH binding leads to the dissociation of the PXR‐HSP90‐CCRP complex in the cytoplasm and translocates PXR into the nucleus, where it interacts with RXR. The ligand‐PXR‐RXR complex binds to DNA response elements, resulting in ALAS1 and CYP450 transcription.^10^, ^13^ In fact, in addition to PXR, there are numerous factors that affect the accumulation of PPIX, such as RXR, ALAS1, CYP3A4, and FECH. The polymorphism effect of SNPs in NR1I2 was sufficiently weak that it may not have a notable effect on ATDH. Furthermore, the interaction effect between SNPs in NR1I2 and other genes or environmental exposure may be observed for ATDH susceptibility. Of course, we must also note that the sample size included in the present meta‐analysis study is relatively low. Although adjusted ORs and 95% CIs were used to evaluate the pooled effect, the covariates adjusted by different studies are not consistent. All of these factors may affect the results of this meta‐analysis, and studies with larger sample sizes are required to assess the association between NR1I2 gene polymorphism and ATDH susceptibility more comprehensively.

In this study, for the first time, we gathered all published articles regarding NR1I2 genetic polymorphisms and the risk of ATDH and increased the sample size to achieve more accurate results. However, this meta‐analysis had several limitations. First, the included studies were limited, and small sample sizes limit the power of analysis results. Moreover, the limited number of studies hindered the adequate exploration of the source of heterogeneity by subgroup analysis. Second, all studies included in this meta‐analysis were identified from selected databases, and publication bias may have occurred. Since fewer than nine studies were included, a publication bias test was not performed. Finally, there is a lack of clarity on some of the issues regarding the uniform criteria used for the diagnosis of ATDH and causality assessment, which may have impacted the true summary effect.

## 5 CONCLUSION

Based on the present meta‐analysis, we did not detect any association between NR1I2 gene polymorphisms and ATDH susceptibility. However, this conclusion should be interpreted with caution due to the low number of studies and the relatively small sample size. More investigation on the association between ATDH and NR1I2 gene polymorphisms is warranted to obtain a reliable conclusion.

## ETHICS APPROVAL

Not required.

## DISCLOSURES

The authors declare that they have no competing interests.

## AUTHOR CONTRIBUTIONS

MMY and YLQ searched the literature, analyzed the data, and wrote the manuscript. YYJ and WPL searched the literature, and extracted data from the collected literature. QLW, HGY, and SWT made substantial contributions to the conception and design of the study and revised the manuscript. All authors approved the final version of the manuscript.

## Supporting information

Supplementary Material

## Data Availability

All data generated and analyzed in the study are available from the corresponding author upon reasonable request.
