# Supplementary material for: NR1I2 genetic polymorphisms and the risk of anti‐tuberculosis drug‐induced hepatotoxicity: A systematic review and meta‐analysis
Source: Pharmacol Res Perspect. 2020 Dec 10;8(6):e00696. doi: 10.1002/prp2.696 (PMC7726956; doi:10.1002/prp2.696)
Supplement: Supplementary file 1 — Supplementary Material [file PRP2-8-e00696-s001.docx]

Table S1. Search strategy for five electronic databases

| Databases | Search strategy | Records |
| --- | --- | --- |
| Pubmed | ((("PXR" or "NR1I2" or "pregnane X receptor" or "nuclear receptor subfamily 1 group I member 2") ) AND ("drug-induced liver injury" or "drug-induced hepatotoxicity" or "drug-induced hepatitis" or "drug-induced liver damage" or "drug-induced hepatic injury" or "toxic hepatitis")) AND ("antituberculosis" or "anti-tuberculosis" or "antitubercular" or "tuberculosis treatment") | 14 |
| Medline | ("PXR" or "NR1I2" or "pregnane X receptor" or "nuclear receptor subfamily 1 group I member 2") AND ("drug-induced liver injury" or "drug-induced hepatotoxicity" or "drug-induced hepatitis" or "drug-induced liver damage" or "drug-induced hepatic injury" or "toxic hepatitis") AND ("antituberculosis" or "anti-tuberculosis" or "antitubercular" or "tuberculosis treatment") | 64 |
| Cochrane Library | “PXR” OR “NR1I2” OR “pregnane X receptor” OR “nuclear receptor subfamily 1 group I member 2” in All Text AND “drug-induced liver injury” OR “drug-induced hepatotoxicity” OR “drug-induced hepatitis” OR “drug-induced liver damage” OR “drug-induced hepatic injury” OR “toxic hepatitis” in All Text AND “antituberculosis” OR “anti-tuberculosis” OR “antitubercular” OR “tuberculosis treatment” in All Text | 3 |
| Web of Science | TS=(("PXR" OR "NR1I2" OR "pregnane X receptor" OR "nuclear receptor subfamily 1 group I member 2") AND ("drug-induced liver injury" OR "drug-induced hepatotoxicity" OR "drug-induced hepatitis" OR "drug-induced liver damage" OR "drug-induced hepatic injury" OR "toxic hepatitis") AND ("antituberculosis" OR "anti-tuberculosis" OR "antitubercular" OR "tuberculosis treatment") ) | 25 |
| SinoMed | ("PXR"[全部字段] OR "NR1I2"[全部字段] OR "孕烷X受体"[全部字段] OR "核受体亚家族1 I组成员2"[全部字段]) AND ( "药物性肝损伤"[全部字段] OR "药物性肝损害"[全部字段] OR "药物性肝炎"[全部字段] OR "药物性肝毒性"[全部字段]) AND ( "抗结核治疗"[全部字段] OR "抗结核药"[全部字段] ) | 0 |


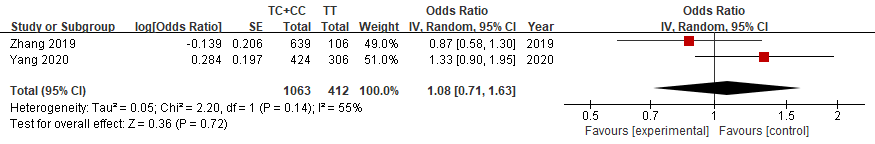


Figure S1. Forest plot of the relation between SNP rs13059232 (dominant model) and the risk of ATDH with the random effects model.


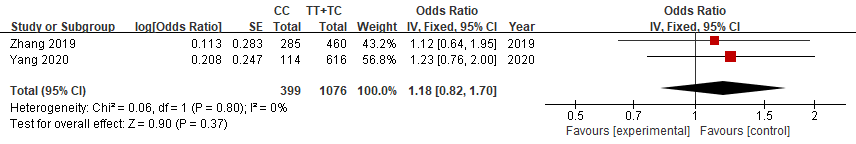


Figure S2. Forest plot of the relation between SNP rs13059232 (recessive model) and the risk of ATDH with the fixed effects model.


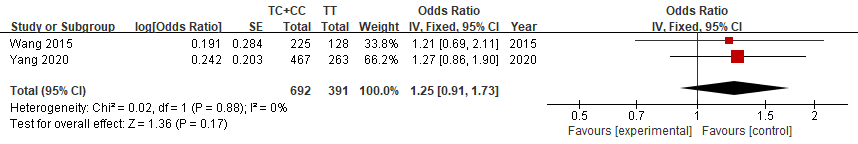


Figure S3. Forest plot of the relation between SNP rs2461823 (dominant model) and the risk of ATDH with the fixed effects model.


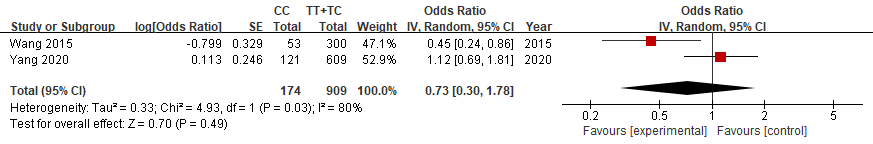


Figure S4. Forest plot of the relation between SNP rs2461823 (recessive model) and the risk of ATDH with the random effects model.


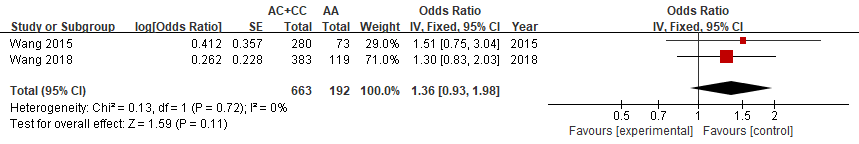


Figure S5. Forest plot of the relation between SNP rs3814057 (dominant model) and the risk of ATDH with the fixed effects model.


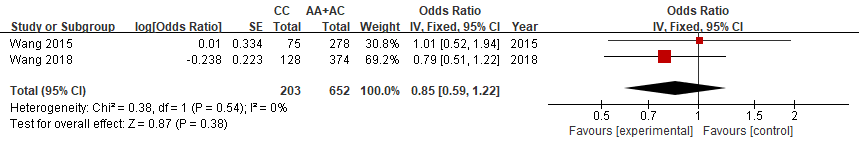


Figure S6. Forest plot of the relation between SNP rs3814057 (recessive model) and the risk of ATDH with the fixed effects model.


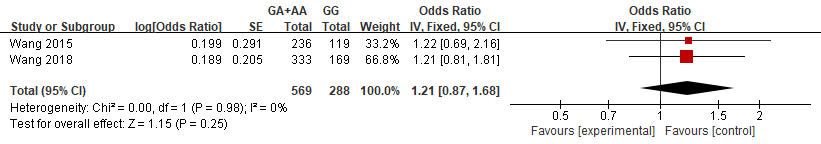


Figure S7. Forest plot of the relation between SNP rs6785049 (dominant model) and the risk of ATDH with the fixed effects model.


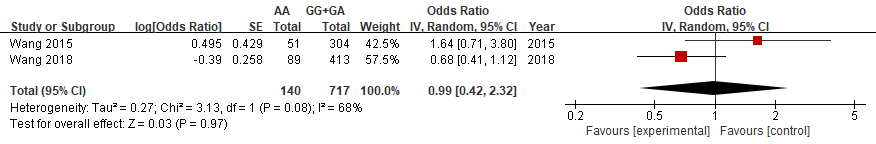


Figure S8. Forest plot of the relation between SNP rs6785049 (recessive model) and the risk of ATDH with the random effects model.
